# The Potential of Essential Oils from Active Packaging to Reduce Ethylene Biosynthesis in Plant Products. Part 1: Vegetables (Broccoli and Tomato)

**DOI:** 10.3390/plants12193404

**Published:** 2023-09-27

**Authors:** Antonio López-Gómez, Alejandra Navarro-Martínez, Alberto Garre, Francisco Artés-Hernández, Pedro Villalba, Ginés Benito Martínez-Hernández

**Affiliations:** 1 Food Safety and Refrigeration Engineering Group, Department of Agricultural Engineering, Universidad Politécnica de Cartagena, Paseo Alfonso XIII, 48, 30203 Cartagena, Murcia, Spain; antonio.lopez@upct.es (A.L.-G.); alejandra.navarro@upct.es (A.N.-M.); pedrovillalbamart@gmail.com (P.V.); 2 Institute of Plant Biotechnology, Campus Muralla del Mar (Universidad Politécnica de Cartagena), 30202 Cartagena, Murcia, Spain; fr.artes-hdez@upct.es; 3 Department of Agricultural Engineering, Universidad Politécnica de Cartagena, Paseo Alfonso XIII, 48, 30203 Cartagena, Murcia, Spain; alberto.garre@upct.es; 4 Postharvest and Refrigeration Group, Department of Agricultural Engineering, Universidad Politécnica de Cartagena, Paseo Alfonso XIII, 48, 30203 Cartagena, Murcia, Spain

**Keywords:** cyclodextrin inclusion complex, plant essential oils, active packaging, ACC oxidase, ACC synthase

## Abstract

Essential oils (EOs) extracted from plants have a high potential to reduce ethylene biosynthesis, although their effects have not been deeply studied yet on the key components of the ethylene biosynthesis pathway: l-aminocyclopropane-1-carboxylic (ACC) oxidase activity, ACC synthase activity, and ACC content. Hence, the present study aimed to elucidate the effects of released EOs from active packaging (with different EO doses ranging from 100 to 1000 mg m^−2^) on the ethylene biosynthesis key components of broccoli and tomato under different storage temperature scenarios. The largest ethylene inhibitory effects on broccoli and tomatoes were demonstrated by grapefruit EO and thyme essential EO (up to 63%), respectively, which were more pronounced at higher temperatures. Regarding EO doses, active packaging with a thyme EO dose of 1000 mg m^−2^ resulted in the strongest reduction (33–38%) of ethylene production in tomatoes. For broccoli, identical results were shown with a lower grapefruit EO dose of 500 mg m^−2^. The studied EO-active packaging decreased ACC synthase and ACC oxidase activities by 40–50% at 22 °C. Therefore, this EO-active packaging is a natural and effective technology to reduce ethylene biosynthesis in broccoli and tomatoes when they are stored, even in unsuitable scenarios at high temperatures.

## 1. Introduction

The alarmingly high global food loss and waste rates require immediate worldwide attention. According to FAO [1] definitions, food loss refers to the early stages of the food supply chain: production, postharvest storage, transportation, and processing. Meanwhile, food waste takes place towards the end of the food supply chain, including retail and consumption [1,2]. In particular, retail and consumption stages related to fruit and vegetables account for 20–40% of the food loss and food waste sum [1]. The United Nations launched an ambitious challenge in 2015 to reduce global food waste per person by half by 2030 as a result of such high rates [3]. The causes of the high waste of fruit and vegetables are mainly related to the reduction in the product quality during its postharvest life due to ripening and senescence processes [2,4]. In particular, ethylene, considered the ripening hormone of plant products, plays a significant role in the postharvest quality reduction of horticultural commodities [5].

The ethylene biosynthesis pathway starts with the conversion of S-adenosyl-L-methionine into l-aminocyclopropane-1-carboxylic acid (ACC) by the enzyme ACC synthase (ACS). Subsequently, ACC can be converted to either 1-(malonylamino)cyclopropane-1-carboxylic acid by the ACC N-Malonyl transferase or to the end product, ethylene, by the ACC oxidase (ACO) [6,7]. In addition, recent investigations have shown evidence of other ACC derivative products (different to ethylene or 1-(malonylamino)cyclopropane-1-carboxylic acid), which are γ-glutamyl-ACC, jasmonyl-ACC, and bacterial metabolization products (α-ketobutyrate and ammonium), as compiled by De Poel et al. [8]. Nevertheless, strong evidence points to ACS and ACO as the two key enzymes of ethylene biosynthesis in plant products [8]. It is well accepted that ACS is the rate-limiting step of ethylene biosynthesis in plants, although there are examples where ACO is the rate-limiting step, e.g., during the post-climacteric ripening of tomato fruit [8].

Groupage of fruit and vegetables during postharvest storage and distribution is a very common practice, which consists of combining different fruit and vegetables in the same place to reduce costs when product quantities are lower. Nevertheless, it must be done with attention since two contradictory situations may converge: (i) horticultural products with high ethylene production and low ethylene sensitivity (e.g., cherry tomatoes); and (ii) horticultural products with low ethylene production but high ethylene sensitivity (e.g., broccoli). Broccoli has very low ethylene production rates (0.5 nmol g^−1^ h^−1^ at 22 °C) [9,10], although it is very sensitive to the ethylene action, resulting in sepal degreening (broccoli yellowing) with ethylene concentrations as low as 10 nL L^−1^ [11]. Nevertheless, ethylene production is higher in tomatoes with values of ≈1.5 μL kg^−1^ h^−1^ in cherry tomatoes (red maturity stages), and ethylene has no significant effects on the cherry tomato colour, although the flesh firmness was reduced [12].

Several technologies have been proposed to reduce the ethylene effects and can be grouped into two major action levels: (i) reduction in ethylene production of the plant product, or (ii) scavenging the produced ethylene from the surrounding atmosphere of the plant product [5,13]. Nevertheless, most of those technologies have a high cost and/or use chemical products, which can be refused by the actual consumer, who is interested in more natural products free of additives in chemical synthesis [14]. Interestingly, plant essential oils (EOs) have a high potential to reduce ethylene production in plant products [10,15,16,17].

Plant EOs are natural extracts with well-known antimicrobial characteristics that are highly valued as natural additives among consumers. Numerous spoilage microorganisms and pathogens are susceptible to the strong in vitro antimicrobial activity of EOs and EO compounds, which has been extensively studied and described in the literature [18]. In addition, EO treatments have been used to maintain the postharvest quality of fruit and vegetables preserving attributes like colour, firmness, etc. [19,20]. The majority of EOs and their primary EO compounds are recognized by the European Union as acceptable food additives. In particular, EO and EO compounds are categorized by the European Union as ‘natural flavouring substances’ and ‘flavouring preparations’, respectively [21,22,23]. However, extra attention must be paid when using high EO doses since distinctive EO off-flavours/odours may be perceived by the consumer in the treated product. However, encapsulation of EOs (e.g., using cyclodextrins) may avoid such sensory disadvantages due to the low but effective EO concentrations released from the EO encapsulation system [24].

Active packaging involves adding active compounds (antimicrobial, antioxidant, or other preservative properties) to the packaging material and subsequent controlled release during the product’s shelf life [25]. Active paper/cardboard packaging with encapsulated EOs (within β-cyclodextrin) is an eco-friendly and cost-effective solution intensively studied and validated at the industrial level by our research group in the last few years, successfully extending the shelf life of fresh fruit and vegetables [16]. The EO release kinetics of such active paper/cardboard packaging technology at different EO doses (100–1000 mg m^−2^) has been fully characterized at different temperatures (2–22 °C range) and relative humidity scenarios (50–60% and 90–95%), which are common during the retail and distribution of fruit and vegetables [24]. In addition, ethylene production was reduced with this EO-active paper/cardboard packaging technology in fruit and vegetables [10,17]. The use of this EO-active packaging technology as an alternative to reduce ethylene production is economically justified since the normal price of a cardboard box is only increased by 3–5%. Nevertheless, a complete study of the effects of the released EOs (from active packaging) has not yet been addressed on the ethylene biosynthesis pathway: ACC, ACO, ACC, and related ethylene production.

This work aimed to study the effect of released EOs, previously encapsulated within β-cyclodextrin, from an active cardboard packaging on the ethylene biosynthesis pathway (ACC, ACO, ACC, and related ethylene production) of broccoli and tomato at different temperatures. Therefore, several active packagings containing different EOs (bergamot EO, grapefruit EO, thyme EO, rosemary EO, linalool, and eugenol) and different doses (100–1000 mg m^−2^) were studied.

## 2. Results and Discussion

### 2.1. ACS Activity

Although the role of ACC in the ethylene biosynthesis pathway has been well characterized since its discovery in 1979 [6], there are still many questions concerning the two proteins associated with ACC in ethylene biosynthesis: ACS and ACO [8]. The ACS can be induced by several factors, like fruit ripening, auxin, flower senescence, disease, and wounding, among others (compiled by Kato et al. [26]). Hence, ACS activity was highly increased in the first 24–36 h after harvest, related to the wounding stress after cutting broccoli stems from the plant [26]. In particular, those authors found a high expression of the ACS-related BO-ACS1 gene in the cutting zone of the broccoli stem.

**Figure 1:**
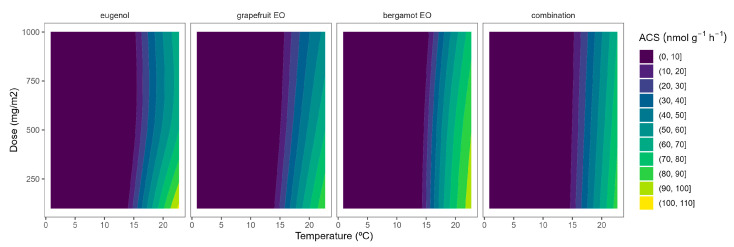
Surface responses for ACS activity of broccoli florets packaged with active packaging including different essential oils (eugenol, bergamot, grapefruit, and their combination) under different temperatures.

The ACS activity of broccoli florets was 175.8 nmol g^−1^ h^−1^ at 22 °C. The ACS activity levels at 22 °C in our study are higher than previously published data [26], which may be explained by the wounding stress (during the 5 h of broccoli floret incubation) after cutting. As expected, the ACS activity was highly reduced at lower temperatures, as observed from the yellow-to-blue ACS turning according to the temperature reduction (Figure 1). In particular, ACS activity levels were reduced to 0.61, 0.38, and 0.21 nmol g^−1^ h^−1^ at 15, 8, and 2 °C, respectively. In that sense, the most interesting inhibitory effects of ACS activity due to EOs released from the active packaging for broccoli, as previously observed for ACO [10], may be observed at 22 °C.

EOs from active packaging induced inhibition of ACS activity (Figure 1). There was a marked effect of the EOs dose on the ACS activity at higher temperatures, as observed in the incipient b-splines curvature at higher temperatures. In particular, the highest EO dose:ACS activity correlation is observed from the higher curvatures for grapefruit EO and bergamot EO at 22 °C. Hence, the ACS activity of broccoli florets at 22 °C was reduced by 27, 37, and 46% when using grapefruit EO-active packaging at 100, 500, and 1000 mg m^−2^, respectively (Figure 1). No remarkable findings were observed related to ACS activity at lower temperatures due to the low enzymatic activity levels at such low temperatures (Figure 1). The effect of EOs on the ACS activity of plant products has not been previously studied to the best of our knowledge.

**Figure 2:**
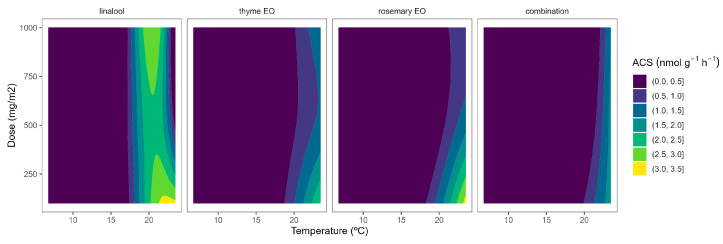
Surface responses for ACS activity of cherry tomatoes packaged with active packaging including different essential oils (linalool, rosemary, thyme, and their combination) under different temperatures (8, 15, and 22 °C).

In tomatoes, the regulation and expression of the ACS and ACO oxidase genes are also under a positive feedback control mechanism, even at the stage of massive ethylene production [27]. Similar to broccoli, the ACS activity of tomato samples was reduced as the temperature increased (Figure 2). In particular, ACS activity values of 5.9, 1.5, and 1.7 nmol g^−1^ h^−1^ were observed at 22, 15, and 8 °C, respectively. These levels are higher than previously published ACS data for tomatoes [28]. It may be explained by the maturity stage and elapsed time after harvest, which are factors highly influencing ACS activity [27,28].

EO-active packaging also induced inhibition of ACS activity in tomatoes (Figure 2). Among EOs, thyme EO and rosemary EO induced the highest dose effects, as observed from the curvature profile at higher temperatures (Figure 2). In particular, thyme EO-active packaging at 100, 500, and 1000 mg m^−2^ induced inhibition of the ACS activity at 22 °C of 33, 53, and 50%, respectively. In that sense, thyme EO-active packaging at 500 mg m^−2^ would be enough to induce similar ACS inhibitory effects in tomatoes as thyme EO-active packaging at higher doses.

### 2.2. ACC Content

**Figure 3:**
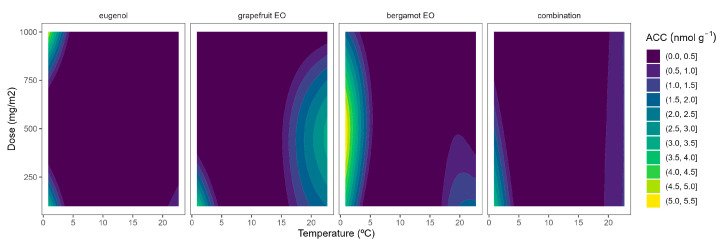
Surface responses for ACC contents of broccoli packaged with active packaging with different doses of inclusion complexes of different essential oils (eugenol, bergamot, grapefruit, and their combination) under different temperatures (2, 8, 15, and 22 °C).

A significant development in our understanding of the process by which ethylene is produced in plants was the discovery of ACC as its precursor, which served as a major building block for other subsequent ethylene biology discoveries [8]. The initial ACC contents of broccoli samples ranged from 2.14 to 6.22 nmol g^−1^, with higher values at higher temperatures (Figure 3). These ACC content levels are slightly higher than the previous literature related to broccoli florets [26]. After excision at harvest, the ACC and ethylene production of broccoli were quickly synthesized in the wounded stem tissue due to the marked increment in ACS activity and enhanced abundance of its transcripts [26]. Accordingly, higher ACC biosynthesis could occur in our broccoli florets due to the wounding effect during broccoli floret preparation.

In general, higher ACC contents were observed as the temperature increased (Figure 3). It may be explained by the observed higher ACS activity at higher temperatures (Figure 1) and consequently higher ACC biosynthesis. In accordance with this, low ACC content variations (less curved lines) were observed at low temperatures as the EOs dose increased, which agrees with the low ACS activity at low temperatures. Among EOs, the combined EO formula showed the most linear behaviour with a low influence of the EOs dose. Similar behaviour was also observed for eugenol at doses higher than 500 mg m^−2^ (Figure 3). Contrary to this, there was a marked EO dose effect for bergamot EO and grapefruit EOs at temperatures over 8 °C. The active packaging effects are more desired at higher storage temperatures when the ethylene biosynthesis is higher. In that sense, the highest ACC reductions were observed for grapefruit EO at 500 and 1000 mg m^−2^ (Figure 3). Other EOs (oregano EO, spearmint EO, cinnamon EO, and carvacrol) were also able to reduce ACC contents in broccoli florets [10].

The initial ACC contents of tomato control samples were approximately 0.5 nmol g^−1^, without significant differences among temperatures (Supplementary File S1). No good data fitting was observed for the ACC data on tomatoes being presented as tabulated data in Supplementary File S1. The ACC contents at 22 °C were similar to those at lower temperatures, which may not be explained by the high ACS activity at 22 °C. This may be due to a higher ACC-ethylene conversion due to the higher ACO activity of tomatoes at 22 °C (see Section 2.3).

In general, active packaging with thyme EO showed an inhibitory effect on the ACC synthesis at all storage temperatures, with values of 0.30, 0.39, and 0.27 nmol g^−1^ for the dose of 1000 mg m^−2^ at 8, 15, and 22 °C, respectively (Supplementary File S1). These findings are in line with the ethylene production data (see Section 2.4). Interestingly, the combined EOs formula showed the lowest ACC contents (0.18–0.20 nmol g^−1^) at 15 °C, although it was punctual only for that temperature due to the lower ACS activity.

### 2.3. ACO Activity

ACO is the second enzyme involved in ethylene biosynthesis, which turns ACC into ethylene when oxygen is present. Contrary to ACS, the post-translational regulation and combinatorial interactions of ACO are far less well understood than those of ACS. It is unclear whether ACO and ACS have similar levels of structural, biochemical, and post-translational complexity because genetic data on ACO are lacking [8]. After harvest, ACO activity significantly increases in broccoli florets, simultaneously with increments in the abundance of BO-ACO1 and BOACO2 transcripts, followed by a marked rise in ethylene production [26].

**Figure 4:**
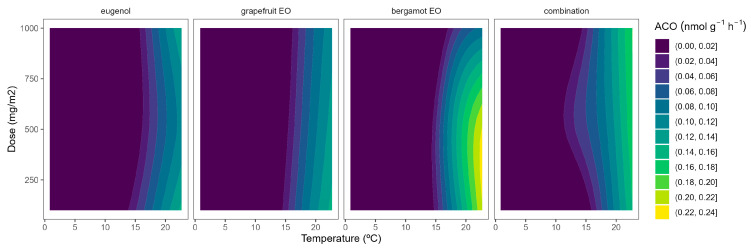
Surface responses for ACO activity of broccoli packaged with active packaging with different doses of inclusion complexes of different essential oils (eugenol, bergamot, grapefruit, and their combination) under different temperatures (2, 8, 15, and 22 °C).

The highest ACO activity increased as the temperature did (Figure 4). In particular, the ACO activity of control samples at 22 °C of 0.6 nmol g^−1^ h^−1^, which agrees with previous data [10,26], was reduced to 0.1–0.3 nmol g^−1^ h^−1^ at lower temperatures. At high temperatures, grapefruit EO showed the highest EO dose:ACO activity curvature among EOs (Figure 4). Hence, grapefruit EO led to ACO activity reductions of 40–45%, compared with control samples, without significant differences among EO doses. We also previously found that other EOs (oregano EO, spearmint EO, cinnamon EO, and carvacrol) inhibited the ACO activity of broccoli florets [10].

**Figure 5:**
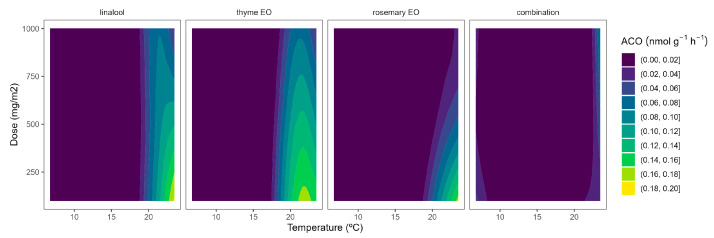
Surface responses for ACO activity of cherry tomatoes packaged with active packaging with different doses of inclusion complexes of different essential oils (linalool, rosemary, thyme, and their combination) under different temperatures (8, 15, and 22 °C).

Although ACS is well accepted as the rate-limiting step of ethylene biosynthesis in plants, there are examples where ACO is the rate-limiting step, e.g., during the post-climacteric ripening of tomato fruit [29]. As previously observed for ACS, the ACO activity was higher at higher temperatures (Figure 5). In particular, ACO activities of 0.4 and 0.1 nmol g^−1^ h^−1^ were observed in control samples at 22 °C and 8/15 °C, respectively. The highest curvatures at high temperatures were observed for rosemary EO, followed by thyme EO. In particular, ACO activity reductions of 47/55/66% and 22/34/50% were observed for rosemary EO and thyme EO, respectively, at 100/500/1000 mg m^−2^, respectively (Figure 5).

### 2.4. Ethylene Production

**Figure 6:**
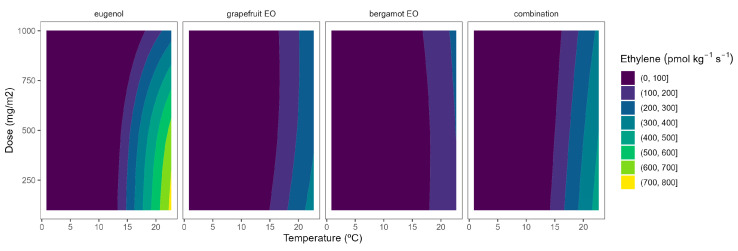
Adjusted surface responses for ethylene production of broccoli packaged with active packaging with different doses of inclusion complexes of different essential oils (bergamot EO, grapefruit EO, eugenol, and their combination) under different temperatures (2, 8, 15, and 22 °C).

**Figure 7:**
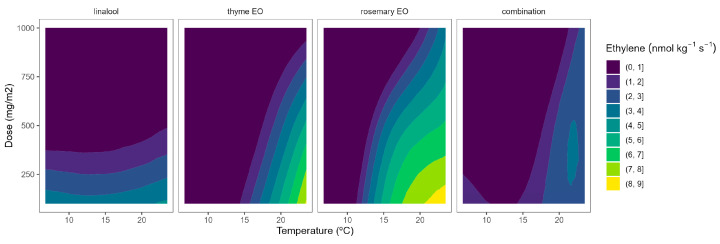
Surface responses for ethylene production of tomato cherry packaged with active packaging with different doses of inclusion complexes of different essential oils (linalool, rosemary, thyme, and their combination) under different temperatures (8, 15, and 22 °C).

The ethylene production in broccoli florets was 34.7, 145.6, 179.9, and 929.1 pmol kg^−1^ s^−1^ at 2, 8, 15, and 22 °C, respectively. Cherry tomatoes showed higher ethylene production with values of 33.8, 35.8, and 41.1 nmol kg^−1^ s^−1^ at 8, 15, and 22 °C, respectively. The obtained data are in accordance with previous literature [9,10,12]. As observed, broccoli is a vegetable with lower ethylene production rates than tomatoes. Though it is highly sensitive to ethylene, the predominant visible result of ethylene action is yellowing, which causes broccoli’s colour to change at ethylene concentrations as low as 10 nL L^−1^ [11]. It has been reported that a shift in the sepal tissue’s sensitivity to the ethylene released by the reproductive structures in the floret mediates the ethylene-induced yellowing of broccoli [11]. On the other side, tomato is a climacteric horticultural product, and its ripening process is accelerated by ethylene, and this endogenous production of that hormone results in a shorter postharvest life [12]. In general, active packaging reduced the ethylene production of broccoli florets and cherry tomatoes by 20–40%, as observed in Figure 6 and Figure 7.

As previously commented, the ethylene production was higher as the temperature increased, turning the b-splines of both vegetables from blue to green (Figure 6 and Figure 7). For broccoli, the ethylene production increases were more related to temperature increments than those related to EO dose ones (Figure 6). In particular, this linear effect was more accurate for grapefruit and bergamot EOs. Using active packaging with a pure EO component (i.e., eugenol) led to a reduction in the width of colour stripes (less abrupture changes) compared to bergamot and grapefruit EOs. It means that temperature increases had a higher correlation with ethylene production changes. Nevertheless, line curvatures became incremental for eugenol as the temperatures augmented, meaning that doses needed to be incremental to reduce the ethylene production increments. An intermediate situation occurred when eugenol was mixed with bergamot and grapefruit EOs.

Overall, ethylene production in broccoli was highly correlated with temperature, although increasing the EO dose of the active packaging did not induce a marked reduction in the ethylene production increments, except for eugenol. Among them, active packaging with bergamot EOs better controlled the ethylene production with bluer lines at the highest temperatures, although no apparent benefits were observed when incrementing the EOs dose. In particular, active packaging with bergamot EOs induced the highest ethylene production control with 56–63% lower values at 22 °C (without significant differences among EOs doses) compared to control samples.

The ethylene production of tomatoes using EO-active packaging showed a different behaviour compared with broccoli (Figure 7). Among EOs, linalool showed a high dose-ethylene production correlation, although it led to a lower temperature-ethylene production correlation. Thyme EO also showed a certain curvature, meaning a positive correlation involving EO dose-ethylene production reduction. Rosemary EO and the triple EO combination showed intermediate situations between linalool and thyme (Figure 7), as observed in Figure 2. Overall, thyme EO-active packaging could be selected, among the rest of the studied EOs, for controlling ethylene production in cherry tomatoes. In particular, thyme EO doses of 1000 mg m^−2^ reduced the ethylene production of tomatoes by 33 and 38% at 8 and 15 °C, respectively (Figure 7). More interestingly, increasing the thyme EO dose from 500 to 1000 mg m^−2^ is supposed to increase the ethylene production control from 22 to 40%.

Zapata et al. [30] also found that supplementation of an edible coating (alginate-based) with EOs (eugenol, thymol, menthol, and carvacrol) reduced by approximately 30–35% the ethylene production of tomatoes during 7 days at 20 °C. Contrary to the reduced literature on vegetables, the inhibitory effect of EOs on the ethylene production of fruits has been widely reported [19,20,31,32,33], although it is very limited to active cardboard packaging with EOs. In particular, Navarro-Martínez et al. [10] reported that the release of EOs (citral, carvacrol, oregano, and cinnamon EOs) from active cardboard packaging reduced the ethylene production of broccoli florets by 30−40% and up to 40−70% during storage of this vegetable at 22 and 2 °C, respectively. Our group has previously studied the effects of released EOs from active cardboard packaging on several fruits, like flat peaches and apples [10,16,17].

## 3. Materials and Methods

### 3.1. Materials

Plant EOs (bergamot, grapefruit, thyme, and rosemary EOs) were acquired from Esencias Martínez Lozano S.A. (Caravaca de la Cruz, Spain). EO composition analyses are included in Supplementary File S2. Eugenol (99.5% purity) and linalool (99.5% purity) were acquired from Merck (Dusseldorf, Germany). β-cyclodextrin, hereinafter referred to as βCD, was provided by Roquette (Kleptose^®^10; Lestrem, France). Water-base lacquer UKAPHOB HR 530 (ammonia-free anionic copolymer; pH 8–10; viscosity max. 100 mPa s at 20 °C; with 30% total solids concentration), which is authorized for food contact surfaces, was acquired from Schill + Seilacher GmbH (Böblingen, Germany). This is a common lacquer type used for paper/cardboard packaging of fruit and vegetables, with the following advantages: (i) it is easily dissolved in water to reach the appropriate density to ensure homogeneous spraying on the cardboard surface; and (ii) when dried, improves the impermeability of the paper/cardboard surface against the high humidity levels maintained (to reduce water loss of plant products) in the cold rooms of horticultural facilities. Recycled kraft paper sheets (50 g cm^−2^) were provided by Bioencapsulation and iPackaging S.L. (Fuente Álamo, Murcia, Spain).

Broccoli (*Brassica oleracea* var. italica) heads were supplied by Sacoje SAT (Lorca, Spain) in May 2022. According to the traceability information consulted by the producer, broccoli heads were manually collected in open-air cultivation parcels in the Southeast Mediterranean Spanish region (Lorca, Spain) at the commercial maturity stage (about 350–400 g per head). Cherry tomatoes (*Solanum lycopersicum* cv. Singular) were purchased in October 2022 from a local producer (Perichán SAT; Cañada de Gallego, Spain). Samples were hand-collected in greenhouses near the producer company in accordance with the traceability information discussed.

### 3.2. Encapsulation of Essential Oils and Active Packaging Preparation

The EOs and EO components used for the active packaging in broccoli (eugenol, bergamot orange EO, grapefruit EO, and their combination in a ratio of 3:1:1 weight(*w*):*w*:*w* corresponding to the major proportion to the EO component) and tomato (linalool, thyme EO, rosemary EO, and their combination in a 3:1:1 *w*:*w*:*w* ratio) were selected based on their high capacity to inhibit the ethylene production in these vegetables based on preliminary experiments with approximately 50 different EOs (and different combinations of them) (data not shown).

The EO−βCD inclusion complex was prepared using the kneading method [34]. Briefly, 1 g of EO was mixed with 7.6 g of βCD (following a 1:1 cavacrol:βCD molar ratio) in a mortar with 3 mL of ethanol, kneaded for 45 min, and finally maintained in a vacuum desiccator at room temperature for at least 72 h (it reduces the surface EO that is not trapped in the βCD cavity). In addition, the characterization (XRD, FTIR, SEM, and TEM) of the EO−βCD inclusion complex of this experiment is included in in Supplementary File S3, showing a high encapsulation efficiency of 86 ± 13%.

The EO−βCD inclusion complex was dissolved (at different concentrations, as subsequently shown) in diluted lacquer before spraying on the kraft paper. The lacquer was previously diluted to a final solid concentration of 8.5% to compensate for the addition of the EO−βCD inclusion complex, as lacquers with a solid content of ≥30% may be difficult to spray on the paper or cardboard surface.

The active packaging was prepared to obtain load levels of the EO−βCD inclusion complex of 100–1000 mg m^−2^, which would be equivalent (based on the previous EOs:βCD molar ratio of 1:7.6) to entrapped 11.6–116.3 mg of EOs per m^2^ of paper. The selected load range of the EO−βCD inclusion complex was selected between the minimum dose to observe the benefits on the product quality during storage and the maximum dose without transferring EO-related off-flavours to the product as previously reported [24]. Active packaging material was prepared one day before the experiments.

### 3.3. Effect of Active Packaging on the Ethylene Biosynthesis System of Vegetables

Tomatoes and broccoli heads were previously sanitised using a NaOCl wash (100 mg L^−1^; 1 min; pH 6.5), followed by a 1-min rinse with tap water. After that, the broccoli was separated into florets. Subsequently, broccoli florets (≈120 g) and tomato fruits (≈230 g) were packaged in rectangular plastic baskets (120 × 110 × 45 mm; 1 L of capacity). A rectangle (120 × 110 mm) of active packaging was placed in the bottom of the basket before being filed with the broccoli florets or tomatoes. Subsequently, trays were thermal-sealed with an automatic packaging machine (Efaman; Efabind, Murcia, Spain) with a Cryovac^®^ EOP616B film (39 µm thickness; Cryovac, Fuenlabrada, Spain) with synthetic air. The gas/water transmission rates of this film were: O_2_, 7.0 cm^3^ m^−2^ day^−1^ atm^−1^; CO_2_, 25.0 cm^3^ m^−2^ day^−1^ atm^−1^; N_2_, 0.5 cm^3^ m^−2^ day^−1^ atm^−1^; water, 10.0 g m^−2^ day^−1^.

Sampling for analyses (ethylene production, ACC content, and ACO and ACS activities) of broccoli was performed after 48, 30, 15, and 5 h of ethylene accumulation at 2, 8, 15, and 22 °C, respectively. For tomatoes, sampling was made after 4, 3, and 2 h at 8, 15, and 22 °C, respectively. The selected accumulation times were based on preliminary tests to achieve measurable ethylene concentrations within packages while avoiding CO_2_ and O_2_ consumption concentrations higher/lower than 5/15% (measured with an O_2_/CO_2_ portable meter; Checkpoint O_2_/CO_2_ model, PBI Dansensor, Barcelona, Spain), respectively, to avoid alteration of the normal product metabolism.

### 3.4. Ethylene Production

The ethylene production was analysed according to previous literature [16]. Briefly, 1 mL of gas was taken from the headspace of the containers using a gas-tight syringe and then injected into a gas chromatograph (GC; Clarus 500 GC; Perkin Elmer Inc., Shelton CT, USA) for the ethylene determination. Two measurements (technical replicates) were made for each basket.

The GC included a GC column (stainless steel column packed with Porapak Q 1/8″, 80/100 mesh size; Teknokroma; Barcelona, Spain), and the analysis conditions were: oven, injector, and flame ionization detector temperatures of 80, 120, and 250 °C, respectively; with synthetic air and H_2_ as gas carriers at 350 and 35 mL min^−1^, respectively. Ethylene content was quantified using an ethylene standard of 10 ppm (gas molar fraction volume) (Praxair; Molina de Segura, Spain). The results were reported as nmol g^−1^ h^−1^.

After collecting samples for the ethylene analyses, the baskets were opened, and the samples were frozen using liquid nitrogen. Then, samples were kept at −80 °C until ACC content and enzymatic analyses (ACO and ACS) were performed.

### 3.5. 1-Aminocyclopropane-1-Carboxylic acid (ACC) Content

The original approach developed by Lizada and Yang [35] (reviewed by Bulens et al. [28]) was used to determine the ACC content. For the ACC extraction, 4 g of ground (IKA A11 Basic; mill with liquid N_2_; Königswinter, Germany) frozen tissue was homogenised (IKA T-18 digital ULTRA-TURRAX^®^; Königswinter, Germany) with 4% salicylic acid solution (in distilled water), followed by vortexing and being placed on ice for 30 min while being stirred. Samples were then centrifuged at 3090× *g* for 10 min at 4 °C. In order to measure the ACC content, 1.4 mL of ACC extract and 0.4 mL of 10 mM HgCl_2_ were combined in a 20 mL glass GC vial before being immediately sealed with an encapsulable septum. After adding 0.2 mL of NaOCl (5% volume (*v*):*v*):NaOH (6 M) mix (2:1 *v:v*) using a syringe, the reaction began. This was followed by an incubation period of 4 min on ice. Ultimately, 1 mL of the GC vial headspace was injected into the GC, and the generated ethylene was analysed (as indicated in Section 3.4). The results were reported as nmol g^−1^.

### 3.6. ACC Oxidase (ACO) Activity

The ACO activity was analysed as described in the literature [28,36]. For the ACO extraction, 0.5 g of ground frozen sample plus 50 mg of polyvinylpolypyrrolidone were added to 1 mL of MOPS (3-morpholinopropane-1-sulfonic acid) buffer (400 mM, pH 7.2), which contained 10% glycerol (*w*:*v*) and 30 mM sodium ascorbate. Then, it was shaken for 15 min at 4 °C. Samples were then centrifuged at 22,000× *g* for 30 min at 4 °C, and the supernatant was used as the ACO extract. The ACO reaction was started by mixing 0.4 mL of the ACO extract with 3.6 mL of ACO reaction buffer (50 mM MOPS buffer comprising 10% glycerol, 1 mM ACC, 10 mM sodium ascorbate, 50 µM iron sulphate, 10 mM sodium bicarbonate, and 1 mM dithiothreitol; pH 7.2) in a 20 mL glass GC vial, which was then rapidly closed with an encapsulable septum. The reaction lasted for 1 h at 30 °C in a water bath. Ultimately, 1 mL of the GC vial headspace was injected into the GC, and the generated ethylene was analysed (as indicated in Section 3.4). The results were reported as nmol g^−1^ h^−1^.

### 3.7. ACC Synthase (ACS) Activity

The ACS activity was analysed as previously described [28,36]. For the ACS extraction, 3 g of ground frozen sample plus 15 mg of polyvinylpolypyrrolidone were added to 3 mL of the extraction buffer, which consisted of 200 mM tricine (pH 8.5) containing 4 μM pyridoxal-L-phosphate and 10 mM dithiothreitol. It was then stirred for 15 min at 4 °C. Samples were then centrifuged at 22,000× *g* for 30 min at 4 °C, and the supernatant was used as the ACS extract. The ACS extract was purified using solid-phase extraction columns (Sephadex G-25 desalting column GE17-0851-01; Sigma Aldrich, Berlin, Germany). For the reaction, 1.5 mL of the purified ACS extract was added to 150 µL of the ACS reaction buffer (tricine buffer 200 mM, pH 8.0) and 150 µL SAM chloride. The reaction continued for 2 h at 25 °C in a water bath. Finally, 200 µL of the 100 mM HgCl_2_ solution was added to stop the reaction. Subsequently, 950 mL of the reacted extract was mixed with 850 mL of distilled water in a 20 mL glass GC vial, which was then quickly sealed with an encapsulable septum. Then, 0.2 mL of the NaOH-NaOCl solution (see Section 3.5) was added through the septum and incubated for 4 min on ice. Ultimately, 1 mL of the GC vial headspace was injected into the GC, and the generated ethylene was analysed (as indicated in Section 3.4). The results were reported as nmol g^−1^ h^−1^.

### 3.8. Data Analysis and Mathematical Modelling

The relationship between the concentration of the measured compounds (ACC, ACO, MACC, ACS, and ethylene) and the packaging conditions (storage temperature, EO dose, and type of antimicrobial) was described using smoothing splines (b-splines) to describe the complex non-linear relationships between these variables. To account more accurately for the experimental error, the model was fitted using Bayesian regression [37].

In practice, the model was implemented in R version 4.2.3 [38] using the *brms* package [39]. For the splines, we use the “t2” implementation included in *mgcv* [40], considering the concentration of the compound as the output variable and the storage temperatures and antimicrobial dose as input variables. The type of antimicrobial (or combination) was introduced as a categorical variable, introducing a random effect on the spline coefficients [41].

The model was fitted using the No-U-Turn sampler included in Stan [42], using the interface provided by *brms*. The convergence of the model was evaluated as is often recommended for this kind of model [37]: visually checking the lack of autocorrelation and appropriate mixing of the trace plots of the Markov chains, ensuring that the R-hat index was lower than 1.01 for every parameter estimate, and checking that the model described the overall trend in the data. The results were represented as a contour plot of the splines using *ggplot2* [43]. The values of the parameter estimates are included in Supplementary File S4.

## 4. Conclusions

This study shows for the first time a detailed scenario of the effects of essential oils (EOs) released from active packaging on the key components of the ethylene biosynthesis system (ACS, ACC, and ACO) of broccoli and tomato. Grapefruit EO and thyme essential EO showed the highest inhibiting effects on the ethylene production of broccoli and tomatoes, respectively, which were more evident at higher storage temperatures. According to the EO doses, the highest inhibition of ethylene production in tomatoes was achieved using active packaging with a thyme EO dose of 1000 mg m^−2^. Meanwhile, similar effects were observed with a lower grapefruit EO dose (500 mg m^−2^) for broccoli. In particular, these EO-active packaging systems reduced ACS and ACO activities by 40–50% at 22 °C. Hence, the studied EO-active packaging systems may greatly reduce the detrimental effects of ethylene on the postharvest quality of these products when they are stored at unrecommended high temperatures. Future studies may deepen the genetic factors involved in the observed inhibition of the key enzymes of the ethylene biosynthesis pathway: ACS and ACO.

## Data Availability

The data presented in this study are available on request from the corresponding author.

## Supplementary Materials

The following supporting information can be downloaded at: https://www.mdpi.com/article/10.3390/plants12193404/s1. Supplementary File S1: ACC content of tomatoes; Supplementary File S2: Composition of the used essential oils; Supplementary File S3: Characterization of the EO-βCD inclusion complex (XRD, FTIR, SEM, and TEM) [44,45]; Supplementary File S4: Statistical analysis files.
