# Supplementary material for: The Potential of Essential Oils from Active Packaging to Reduce Ethylene Biosynthesis in Plant Products. Part 1: Vegetables (Broccoli and Tomato)
Source: Plants (Basel). 2023 Sep 27;12(19):3404. doi: 10.3390/plants12193404 (PMC10574596; doi:10.3390/plants12193404)
Supplement: Supplementary file 1 [file plants-12-03404-s001.zip › Supplementary File S2_Composition of the used essential oils.pdf]

Supplementary File S2. Composition of the used essential oils

Bergamot essential oil

Component	Content (%)
β-pinene	5.5–9.5
Limonene	30-45
γ-Terpinene	6-10
Linalool	3-15
Linalyl acetate	22-36
β-Bisabolene	0.30-0.55
Geranial	0.28–0.50

Grapefruit essential oil

Component	Content (%)
α-pinene	0.2 - 0.6
β-pinene	0.05 - 0.2
Sabinene	0.1 - 0.6
β-myrcene 1-3	1.5 - 2.5
Limonene	92 - 96
Octanal	0.2 - 0.8
Nonanal	0.04 - 0.1
Decanal	0.1 - 0.6
β-caryophyllene	0.2 - 0.5

Thyme essential oil

Component	Content (%)
α-pinene	3-8

β-myrcene	2-6
Limonene	1.5 - 4
1,8-cineole + β -	25 - 35
Camphre	4 - 12
β-caryophyllene	1-3
Neral	0.5 - 3.5
Geranial	0.5 - 2.5
α-terpineol	1.5 - 3.5
Borneol	1.5 - 4.5

Rosemary essential oil

Component	Content (%)
α-pinene + α-thuyene	18 - 26
Camphene	8 - 13
β-myrcene	2.5 - 5
Limonene	2.5 - 5.5
1-8-cineole	17 - 25
p-cymene	1 - 2
Camphre	12.5 - 22
Bornyl acetate	0.4 - 2.5
α-terpineol	1 - 3.5
Borneol	2 - 4
