# Supplementary material for: The Potential of Essential Oils from Active Packaging to Reduce Ethylene Biosynthesis in Plant Products. Part 1: Vegetables (Broccoli and Tomato)
Source: Plants (Basel). 2023 Sep 27;12(19):3404. doi: 10.3390/plants12193404 (PMC10574596; doi:10.3390/plants12193404)
Supplement: Supplementary file 1 [file plants-12-03404-s001.zip › Supplementary File S3_Characterization of the EO-CD inclusion complex.pdf]

Supplementary File S3. Characterization of the EO-CD inclusion complex (XRD, FTIR, SEM, and TEM)

X-Ray diffraction (XRD) analyses of βCD and the EO-βCD inclusion complex are shown in Figure S3.1. The XRD analysis shows that the intense and sharp peaks of the β-CD is crystalline in nature. After EO encapsulation, the intensity and profile of β-CD were modified (e.g. peaks at 4.8 ºC and 8 ºC), which indicates the formation of bonds between the β-CD molecule and EO [1].

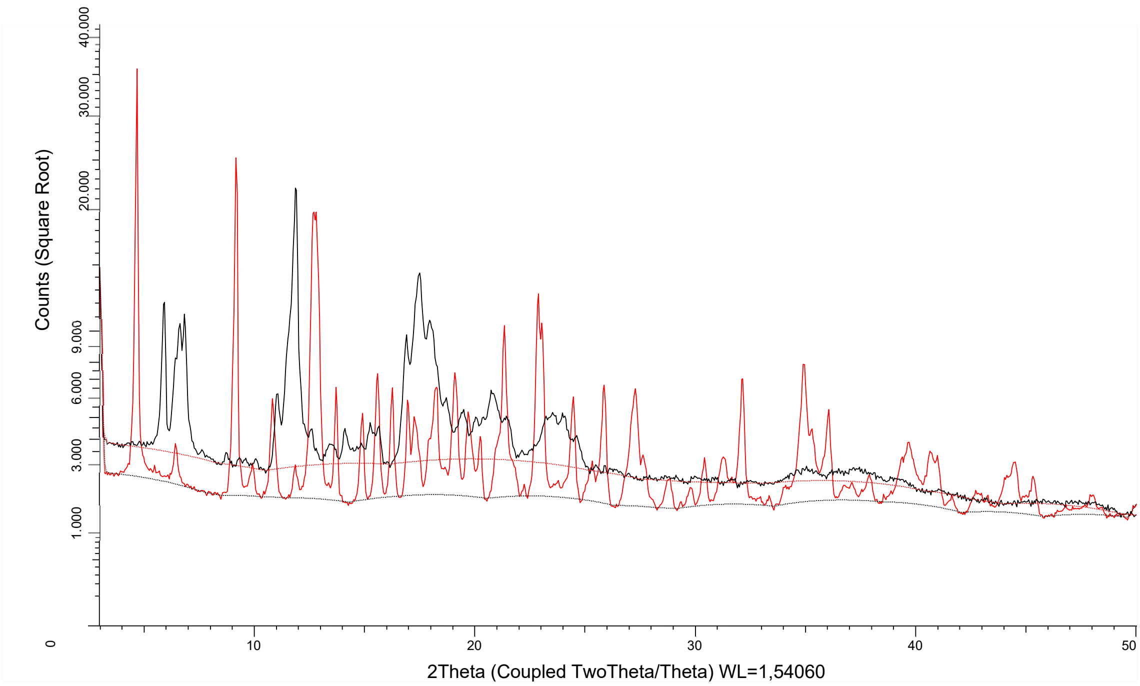

Figure S3.1. X-Ray diffraction (XRD) analyses of β-CD (red colour line) and the EO-βCD inclusion complex (black colour line). An XRD diffractometer (Bruker D8 Advance; Bruker, Billerica MA, USA) was used. X-ray diffractograms were obtained using Cu-Kα ($\lambda = 1.5418$ Å) radiation in a 2θ range from 3° to 60° with 0.02° steps at 1°/min.

Fourier Transform Infrared spectroscopy (FTIR) analyses of βCD and the EO-βCD inclusion complex are shown in Figure S3.2. The characteristic peaks of βCD were identified at 3,300 (−OH stretch), 2,925 (vibration of C−H stretch), 1,643 (bending of H–O–H), 1,157 (vibrations of the asymmetric stretch of the C–O–C) and 1,023 cm^{-1} (symmetric stretching link C–O–C). Nevertheless, the FTIR spectra of the EOs-βCD inclusion complex showed that the intensity of peaks was minimised, which indicates the modification of bonds after the inclusion complex formation, as previously found [1,2].

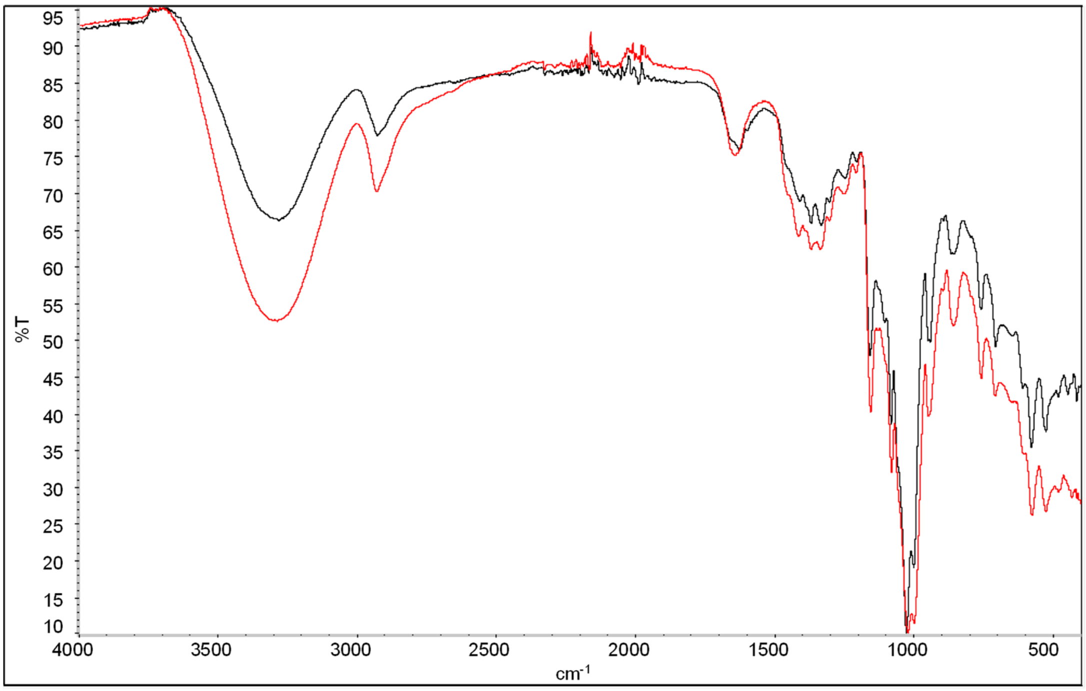

Figure S3.2. Fourier Transform Infrared spectroscopy (FTIR) analyses of β-CD (red colour line) and the EO-βCD inclusion complex (black colour line). An FTIR spectrometer (Thermo Scientific Nicolet 5700, Berlin, Germany) was used in absorbance mode and wavenumbers between 400 and 4,000 cm^{-1}. For sample preparation, 2 mg of samples were mixed with 200 mg of KBr and this mixture was then pressed to form tablets with a thickness of 1 mm.

Scaning electron microscope (SEM) images (Figure S3.3) show that the particle size of the EO-βCD inclusion complex is smaller than the particle size of pure βCD, which may indicate the formation of the inclusion complex.

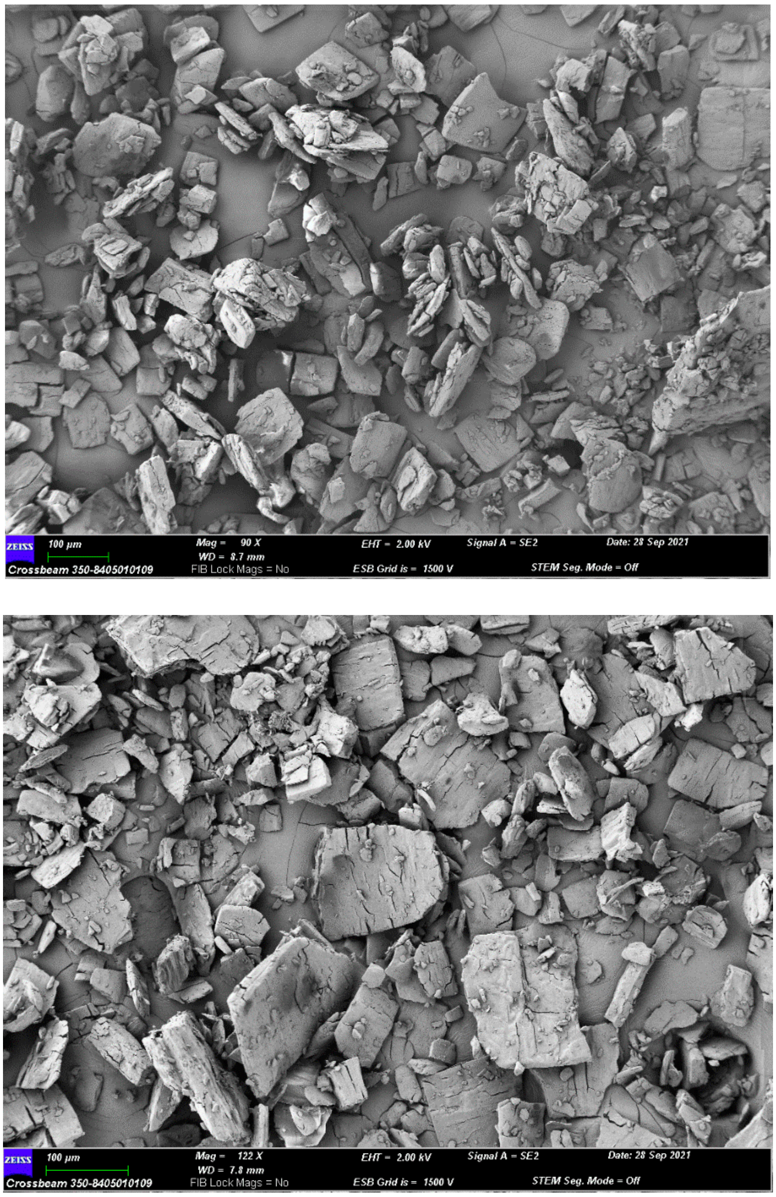

Figure S3.3. The microscopic morphology of β-CD (up image) and the EO-βCD inclusion complex (down image) was studied using a scanning electron microscope (SEM) (S−3500N; Hitachi, Tokyo, Japan). Samples were previously coated with gold in a sputter coater (SC7640; Quorum Technologies, East Sussex, England) and then observed with the SEM at 2 kV.

Transmission electron microscopy (TEM) images (Figure S3.4) show the particle size of the EO-βCD inclusion complex.

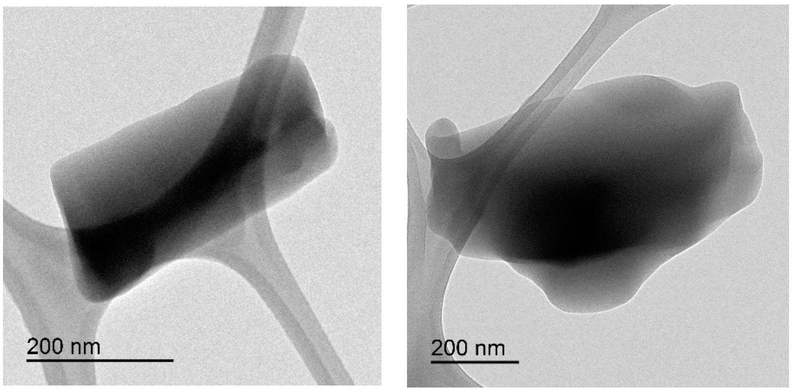

Figure S3.4. Transmission electron microscopy (TEM) of the EO-βCD inclusion complex was studied using a TEM (JEM-1400 Plus; Jeol Ltd., Tokyo, Japan), which worked at 120 keV. Samples were previously dispersed in deionized water using an ultrasonic homogenizer for 5 min. Certain quantity of the suspension was diluted and dropped onto a carbon-coated grid and kept for 1 min. After the removal of excess suspension by filter paper, the grid was dried overnight.

1. Marques, C.S.; Carvalho, S.G.; Bertoli, L.D.; Villanova, J.C.O.; Pinheiro, P.F.; dos Santos, D.C.M.; Yoshida, M.I.; de Freitas, J.C.C.; Cipriano, D.F.; Bernardes, P.C. β-Cyclodextrin inclusion complexes with essential oils: Obtention, characterization, antimicrobial activity and potential application for food preservative sachets. *Food Res. Int.* **2019**, *119*, 499–509, doi:10.1016/j.foodres.2019.01.016.
2. Buendía−Moreno, L.; Soto−Jover, S.; Ros−Chumillas, M.; Antolinos−López, V.; Navarro−Segura, L.; Sánchez−Martínez, M.J.; Martínez−Hernández, G.B.; López−Gómez, A. An innovative active cardboard box for bulk packaging of fresh bell pepper. *Postharvest Biol. Technol.* **2020**, *164*, 111171, doi:10.1016/j.postharvbio.2020.111171.
